# Metabolomic Evaluation of *Scenedesmus* sp. as a Feed Ingredient Revealed Dose-Dependent Effects on Redox Balance, Intermediary and Microbial Metabolism in a Mouse Model

**DOI:** 10.3390/nu11091971

**Published:** 2019-08-21

**Authors:** Yiwei Ma, Wenguang Zhou, Paul Chen, Pedro E. Urriola, Gerald C. Shurson, Roger Ruan, Chi Chen

**Affiliations:** 1 Department of Food Science and Nutrition, University of Minnesota, St. Paul, MN 55108, USA; 2 Department of Bioproducts and Biosystems Engineering, University of Minnesota, St. Paul, MN 55108, USA; 3 Department of Animal Science, University of Minnesota, St. Paul, MN 55108, USA

**Keywords:** *Scenedesmus*, microalgae, growth performance, exposure markers, redox balance, lipidome, purine metabolism, microbial metabolism

## Abstract

*Scenedesmus* is a common green algae genus with high biomass productivity, and has been widely used in biofuel production and waste water management. However, the suitability and metabolic consequences of using *Scenedesmus* as an animal feed ingredient have not been examined in detail. In this study, the influences of consuming *Scenedesmus* on the metabolic status of young mice were investigated through growth performance, blood chemistry, and liquid chromatography-mass spectrometry (LC-MS)-based metabolomics. Compared to the control diet, feeding a diet containing 5% *Scenedesmus* improved growth performance while the diet containing 20% *Scenedesmus* suppressed it. Among common macronutrients-derived blood biochemicals, serum triacylglycerols and cholesterol levels were dramatically decreased by feeding the 20% *Scenedesmus* diet. Metabolomic analysis of liver, serum, feces, and urine samples indicated that *Scenedesmus* feeding greatly affected the metabolites associated with amino acid, lipid, purine, microbial metabolism, and the endogenous antioxidant system. The growth promotion effect of feeding the 5% *Scenedesmus* diet was associated with elevated concentrations of antioxidants, an expanded purine nucleotide cycle, and modified microbial metabolism, while the growth suppression effect of feeding the 20% *Scenedesmus* diet was correlated to oxidative stress, disrupted urea cycle, upregulated fatty acid oxidation, and an imbalanced lipidome. These correlations among *Scenedesmus* dietary inclusion rate, individual metabolite markers, and growth performance suggest the need to define the dietary inclusion rate threshold for using *Scenedesmus* and other microalgae supplements as feed ingredients, and also warrant further mechanistic investigations on the biological processes connecting specific constituents of *Scenedesmus* with the metabolic effects observed in this study.

## 1. Introduction

Microalgae are more efficient than many agricultural crops in harvesting solar energy and fixing carbon dioxide for nutrient production. This outstanding autotrophic property has led to extensive exploration of microalgae as a staple source of human food in the 1940s and 1950s [1]. However, the effort was mostly abandoned in the 1960s due to both the technical challenges and economic sustainability posed by large-scale microalgae farming [2]. Despite this setback, microalgae continues to be a common source of dietary supplements for humans and animals because of their valuable nutrient composition, including essential amino acids, vitamins, minerals, polyunsaturated fatty acids (PUFAs) [3], and other bioactive constituents, such as pigments and fiber [4,5]. These nutrients can support energy metabolism and nutrient utilization efficiency in humans and animals, while the bioactive constituents provide antioxidant, anti-inflammatory, and hypolipidemic benefits [6].

*Scenedesmus* is a large genus of green microalgae that contains many common freshwater species [7]. Known for their efficiency in photosynthesis and their capacity for lipid accumulation and nutrient removal from the environment, *Scenedesmus* species, together with other green microalgal species, have been widely used for biodiesel production and waste water treatment [8,9]. Considering their diverse nutrient and bioactive constituents, *Scenedesmus* biomass can be expected to cause changes as well as pose challenges in the metabolic system when fed to animals. However, current knowledge on the metabolic effects of feeding diets containing *Scenedesmus*, as well as feeding other microalgae species, has been limited to targeted analysis of growth performance and the blood biochemical parameters related to lipids and redox balance [4,6,10]. A comprehensive investigation on the metabolic effects of feeding *Scenedesmus* has not been reported.

In the current study, an untargeted examination of *Scenedesmus*-elicited metabolic effects was conducted through the liquid chromatography-mass spectrometry (LC-MS)-based metabolomic analysis of serum, hepatic extract, fecal extract, and urine samples from young mice fed dried *Scenedesmus* powder. The metabolites associated with dose-dependent effects of *Scenedesmus* feeding were identified and further characterized for their correlations with microalgal constituents and animal performance.

## 2. Materials and Methods

### 2.1. Culture of Scenedesmus Algae

*Scenedesmus* sp. (UMN 258), is a species isolated locally in Minnesota, and was chosen as the source of green microalgae in our mouse feeding experiment because of its favorable nutrient composition. Our previous screening study has shown that UMN 258 had a much higher concentration of eicosapentaenoic acid (EPA) than other examined local green microalgae species [11]. The cultivation of UMN 258 was initiated by inoculating its seeds into autoclaved BG-11 medium containing 2 g/L glucose, and then maintained at 25 ± 2 °C on a shaker at 100 rpm under a continuous cool-white fluorescent light illumination at 100 μmol/m^2^/s. After 1 to 2 weeks of batch cultivation, *Scenedesmus* biomass was separated from the culture broth by centrifugation at 500× *g* for 10 min and washed with deionized water. After repeating this centrifugation–washing process twice, harvested *Scenedesmus* biomass was dried and stored at 4 °C prior to the feeding experiment [12].

### 2.2. Chemicals

The sources of reagents and standards are listed in Supplementary Table S1.

### 2.3. Animal Treatment and Sample Collection

Male C57BL/6 mice (*n* = 24), 8 weeks old, were purchased from Charles River Lab (Wilmington, MA). All mice were housed in the University of Minnesota animal facility at a constant temperature of 21 °C under a 12 h light–dark cycle, and had access to water and feed ad libitum. Handling and treatment procedures were used in accordance with animal study protocols approved by the UMN Institutional Animal Care and Use Committee. Mice were first acclimated to the control AIN93G diet (CTL) for 7 days, and then randomly divided into three feeding groups (8 mice/group), CTL, 5% *Scenedesmus* diet, and 20% *Scenedesmus* diet. The 5% and 20% experimental diets were prepared by mixing AIN93G and dried *Scenedesmus* powder, in 95:5 (w/w) and 80:20 (w/w) ratios, respectively. The nutrient content in three formulated diets met or exceeded the National Research Council (NRC) requirements (2012) for mice (Supplementary Table S2). On day 26 of the feeding experiment, urine and fecal samples were collected by housing animals individually in the metabolic cages for 24 h. On day 28, blood samples were collected by submandibular bleeding, and tissue samples were collected after carbon dioxide euthanization. All samples were stored at −80 °C until further analysis.

### 2.4. Growth Performance Measurement

Mice were weighed individually every day in the first 7 days of feeding experimental diets, and then 3 times weekly during the following 3 weeks to monitor their health and calculate average daily body weight gain (ADG). On each weighing day, the amount of food disappearance was measured to calculate average daily food intake (ADFI). Cage ADG and ADFI were used to calculated gain:feed ratio (G:F ratio).

### 2.5. Serum Biochemical Analysis

Serum cholesterol, triacylglycerols (TAG), glucose, and blood urea nitrogen (BUN) concentrations were determined using respective colorimetric assay kits from Pointe Scientific (Canton, MI, USA) in a 96-well plate reader (SpectraMax 250, Molecular Devices, Sunnyvale, CA, USA).

### 2.6. Synthesis of Glycol-4-Methyl Pentanoic Acid and Glycol-4-Methyl Hexanoic Acid

To synthesize the glycine conjugates, 4-methyl pentanoic acid or 4-methyl hexanoic acid was first dissolved in thionyl chloride and incubated at 80 °C for 30 min to form respective fatty acid chlorides, which were then added into a glycine suspension in dry pyridine for the conjugation reaction. The reaction mixture was incubated at 4 °C for 24 h with continuous stirring, and then acidified with 80 µL of 12 M HCl. After 10 min centrifugation at 18,000× *g*, the supernatant was collected and diluted 1000 times for the structural confirmation in the LC-MS analysis.

### 2.7. Sample Preparation for LC-MS Analysis

Urine samples were mixed with 5 volumes of 50% aqueous ACN and centrifuged at 18,000× *g* for 10 min to remove the proteins and particles. Fecal samples were soaked in 50 volumes (v/w) of 50% aqueous ACN overnight at 4 °C, extracted by vortexing and sonication for 10 min, and centrifuged at 18,000× *g* for 10 min to remove the insoluble fraction. Serum samples were mixed with 19 volumes of 66% aqueous ACN, and then centrifuged at 18,000× *g* for 10 min to obtain the supernatants. Liver extracts were prepared based on the principle of Bligh and Dyer method [13]. Briefly, 100 mg of frozen liver sample was homogenized in a mixture of 0.5 mL methanol, 0.5 mL chloroform, and 0.4 mL water. After 10 min centrifugation at 18,000× *g*, the top aqueous fraction and the bottom organic fraction were separated. The organic fraction was dried under nitrogen and reconstituted in 0.5 mL n-butanol. All samples were stored at −80 °C for further analysis.

### 2.8. Chemical Derivatization

For detecting the metabolites containing amino functional group, samples were derivatized with dansyl chloride (DC) prior to the LC-MS analysis. Briefly, 5 µL of sample or standard was mixed with 5 µL of 100 µM *p*-chlorophenylalanine (internal standard), 50 µL of 10 mM sodium carbonate, and 100 µL of DC (3 mg/mL in acetone). The mixture was incubated at 60 °C for 15 min and centrifuged at 18,000× *g* for 10 min. The supernatant was transferred into a HPLC vial for LC-MS analysis. For detecting carboxylic acids, aldehydes and ketones, the samples were derivatized with HQ prior to the LC-MS analysis [14]. Briefly, 2 µL of sample was added into a 100 µL of freshly-prepared ACN solution containing 10 mM DPDS, 10 mM TPP, and 10 mM HQ. The reaction mixture was incubated at 60 °C for 30 min, followed by chilling on ice and mixing with 100 µL of ice-cold H_2_O. After centrifugation at 18,000× *g* for 10min, the supernatant was transferred into a HPLC vial for LC-MS analysis.

### 2.9. LC-MS Analysis

A 5 µL aliquot of diluted urine, fecal, serum, or liver aqueous fraction samples was injected into an Acquity ultra-performance liquid chromatography (UPLC) system (Waters, Milford, MA, USA) and separated in a BEH C18 column with a gradient of mobile phase ranging from water to 95% aqueous acetonitrile consisting of 0.1% formic acid in a 10 min run. The LC eluate was directly introduced into a Xevo-G2-S QTOF mass spectrometer for the accurate mass measurement and ion counting. Capillary voltage and cone voltage for electrospray ionization (ESI) was maintained at 3 kV and 30 V for positive-mode detection, or at −3 kV and −35 V for negative-mode detection, respectively. Nitrogen was used as both cone gas (50 L/h) and desolvation gas (600 L/h) and argon as collision gas. For accurate mass measurement, the mass spectrometer was calibrated with sodium formate solution (range *m/z* 50–1000) and monitored by the intermittent injection of the lock mass leucine encephalin ([M + H]^+^ = *m/z* 556.2771 or [M + H]^−^ = *m/z* 554.2615). Mass chromatograms and mass spectral data were acquired and processed by MassLynx^TM^ software (Waters, Milford, MA, USA) in centroided format. Additional structural information was obtained by tandem MS (MS/MS) fragmentation with collision energies ranging from 15 to 45 eV.

### 2.10. Multivariate Data Analysis

Chromatographic and spectral data of samples were analyzed using MarkerLynx software (Waters). A multivariate data matrix containing information on sample identity, ion identity (retention time (RT) and *m/z*), and ion abundance was generated through centroiding, deisotoping, filtering, peak recognition, and integration. The intensity of each ion was calculated by normalizing the single ion counts (SIC) versus the total ion counts (TIC) in the whole chromatogram. The data matrix and sample list were further exported into SIMCA-P+ software (Umetrics, Kinnelon, NJ, USA). Principal components analysis (PCA) and projection to latent structures-discriminant analysis (PLS-DA) were generated after data were transformed by mean-centering and Pareto optimization to analyze the data from control and *Scenedesmus*-treated C57BL/6 mice. Major latent variables in the data matrix were described in a scores scatter plot of multivariate model. The potential metabolites after *Scenedesmus* feeding were identified by analyzing ions contributing to the principal components and to the separation of sample groups in the loadings scatter plot.

### 2.11. Marker Characterization and Quantification

The chemical identities of compounds of interest were determined by accurate mass measurement, elemental composition analysis, database search (Human Metabolome Database: http://www.hmdb.ca/, Lipid Maps: http://www.lipidmaps.org/, Metlin: https://metlin.scripps.edu/), MSMS fragmentation, and comparisons with authentic standard if available. The concentrations of selected metabolites were determined by calculating the ratio between the peak area of metabolite and the peak area of internal standard, and fitting with a standard curve using QuanLynx^TM^ software (Waters). The distribution of metabolite markers in sample groups was present in relative levels or in heatmaps. The relative level of individual metabolite (fold) was calculated as the ratio between its mean abundance in a specific sample group and its highest mean abundance in all sample groups. The heatmap of selected metabolite markers was generated by the heatmap.2 package in R program after the Z score transformation of relative abundances (http://www.R-project.org), and the correlations among these metabolite markers were defined by hierarchical clustering analysis (HCA).

### 2.12. Gene Expression Analysis

Total RNA from the liver samples was extracted using TRIzol reagent, and cDNA was generated from 1 µg of total RNA using SuperScript III Reverse Transcriptase (Thermo Fisher Scientific, Waltham, MA, USA). The sequences of the primers used for the quantitative real-time polymerase chain reaction (qPCR) analysis of targeted genes, including acyl-CoA thioesterase 1 (*Acot1*), carnitine palmitoyltransferase 1 (*Cpt1*), phosphatidylethanolamine *N*-methyltransferase (*Pemt*), glutamate-cysteine ligase modifier subunit (*Gclm*), glutamate-cysteine ligase catalytic subunit (*Gclc*) were designed using the NCBI/Primer-BLAST tool (Supplementary Table S3). The qPCR reactions were conducted using the SYBR green PCR master mix (Thermo Fisher Scientific) in a StepOnePlus system (Applied Biosystems, CA, USA). Using β-actin as the reference gene, the expression levels of genes were quantified using the comparative threshold cycle (CT) method.

### 2.13. Statistical Analysis

Statistical analysis were performed using GraphPad Prism 6 software (GraphPad Software, Inc., La Jolla, CA, USA). Data are presented as mean ± SEM. Homogeneity of variance for ANOVA was assessed by Bartlett’s test. The one-way ANOVA using dietary treatment as the fixed factor was performed. For metabolomics data, one-way ANOVA was conducted on the original abundances of individual metabolite instead of the relatives to highest level values. The Tukey post hoc test was performed if the one-way ANOVA was significant. Differences were considered significant if *P* < 0.05.

## 3. Results

### 3.1. Growth Responses to Scenedesmus Feeding

**Figure 1:**
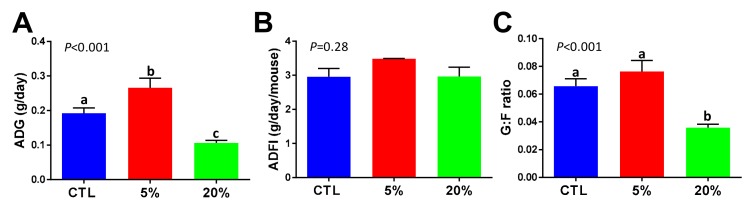
Influences of *Scenedesmus* feeding on growth performance. The body weight gain and feed intake of three groups of mice, control (CTL), 5% *Scenedesmus*, and 20% *Scenedesmus*, were monitored during a 28-day feeding period. (**A**) Average daily gain (ADG). (**B**) Average daily food intake (ADFI). (**C**) Gain to feed ratio (G:F). *P*-values indicate overall significances across all sample groups from the one-way ANOVA test. Means with different letter labels (a,b,c) indicate significant differences (*P* < 0.05) between two dietary treatments by the Tukey post hoc test.

Young mice in their growing phase are generally considered to be sensitive to dietary nutrient content [15]. The C57BL/6 mice used in this study were eight weeks of age prior to the feeding of CTL, 5% *Scenedesmus*, and 20% *Scenedesmus* experimental diets. After 28 days of feeding, significant differences in growth performance were observed among the three treatment groups of mice. Compared to the control group, feeding the 5% *Scenedesmus* diet increased the ADG, whereas feeding the 20% *Scenedesmus* diet decreased it (Figure 1A). There was no effect of dietary treatment on ADFI (Figure 1B). The gain efficiency, indicated by G:F ratio, was decreased by feeding the 20% *Scenedesmus* diet (Figure 1C). Furthermore, 20% *Scenedesmus* feeding might have caused polyuria, because the volume of 24 h urine produced by this group of mice was two to three folds greater than other two groups. Overall, these observations suggested that feeding 5% *Scenedesmus* promoted the growth, while 20% *Scenedesmus* was associated with negative responses.

### 3.2. Effect of Scenedesmus Feeding on Blood Chemistry

**Figure 2:**
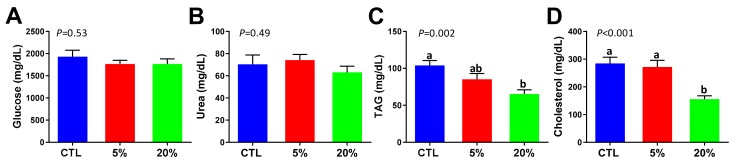
Influences of *Scenedesmus* feeding on blood biochemical analysis. (**A**) Serum glucose concentration. (**B**) Serum blood urea nitrogen (BUN) concentration. (**C**) Serum triacylglycerols (TAG) concentration. (**D**) Serum cholesterol concentration. *P*-values indicate overall significances across all sample groups from the one-way ANOVA test. The means with different letter labels (a,b) indicate significant differences (*P* < 0.05) between two dietary treatments by the Tukey post hoc test.

Serum glucose, BUN, TAG, and cholesterol concentrations partially reflect the general status of macronutrient metabolism. The results from blood chemistry analysis showed that neither the 5% nor the 20% *Scenedesmus* diets affected the serum concentrations of glucose and BUN (Figure 2A,B). However, after four-week feeding of the 20% *Scenedesmus* diet, serum TAG and cholesterol concentrations became lower than that of the control group (Figure 2C,D).

### 3.3. Metabolomic Investigation of Scenedesmus-Induced Metabolic Events

**Figure 3:**
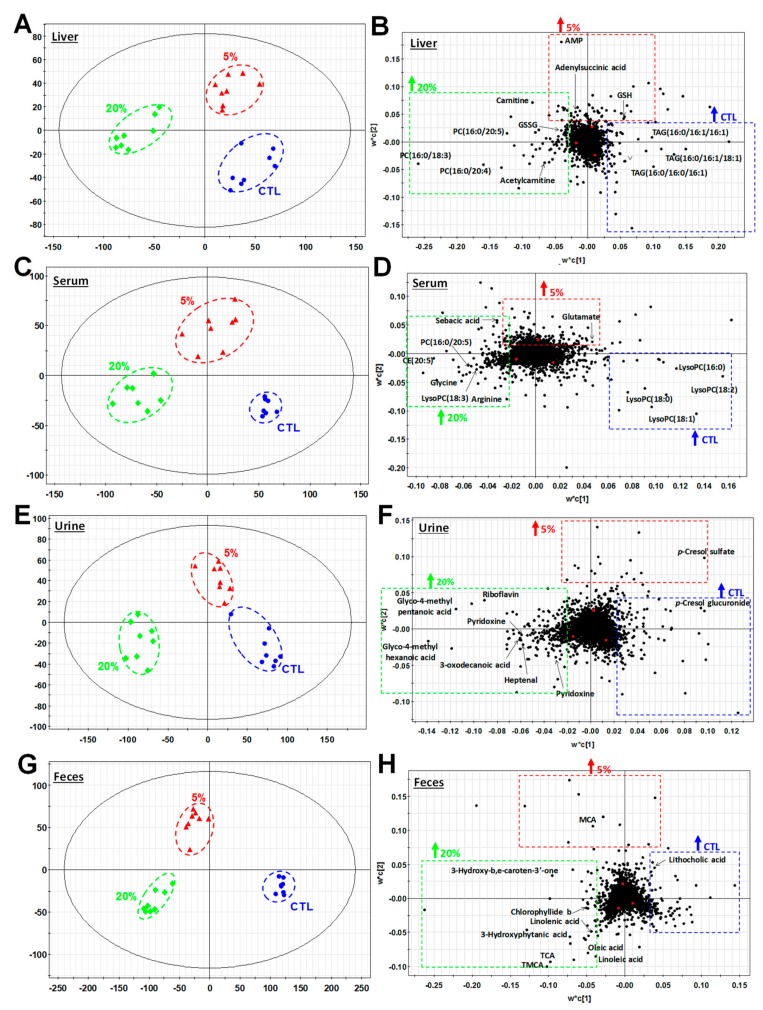
Effects of *Scenedesmus* diets on hepatic, serum, urinary, fecal metabolomes. Data from LC-MS analysis of liver extract, serum, urine and fecal extract were processed by supervised partial least squares-discriminant analysis (PLS-DA) modeling. The associations of metabolites representing each of the three groups of mice (*n* = 8/group) are shown in the scores plots. Selective metabolite markers correlating with the experimental diets are labeled in the loadings plots. (**A**) Scores plot of a PLS-DA model on liver extracts. (**B**) Loadings plot of a PLS-DA model on liver extracts. (**C**) Scores plot of a PLS-DA model on serum samples. (**D**) Loadings plot of a PLS-DA model on serum samples. (**E**) Scores plot of a PLS-DA model on urine samples. (**F**) Loadings plot of a PLS-DA model on urine samples. (**G**) Scores plot of a PLS-DA model on fecal samples. (**H**) Loadings plot of a PLS-DA model on fecal samples. The symbol “↑” in the loadings plot indicates positive correlation with individual treatment.

*Scenedesmus*-induced metabolic events were examined through the LC-MS-based metabolomic analysis of liver, serum, urine, and fecal extracts of samples obtained from mice fed the control, 5%, and 20% *Scenedesmus* diets. Dose-dependent separation of metabolites from the three feeding groups were observed in the scores plots of four PLS-DA models on these four types of samples, respectively, suggesting that *Scenedesmus* feeding altered the mouse metabolome in a dose-dependent pattern (Figure 3A,C,E,G). More importantly, the separation between control and 20% *Scenedesmus* treatments mainly occurred along the first principal component of the PLS-DA models, while the 5% *Scenedesmus* treatment differed from the control and 20% *Scenedesmus* treatment mainly in the second principal component (Figure 3A,C,E,G). This pattern of sample distribution in the PLS-DA models suggests that 5% and 20% *Scenedesmus* diets had different influences on the mouse metabolome. Subsequently, the metabolites positively associated with individual dietary treatment was identified in the corresponding loadings plots of PLS-DA models (Figure 3B,D,F,H) based on the correlation analysis (Supplementary Table S4). Their chemical identities were then defined by elemental composition analysis, database search, MSMS fragmentation, and confirmation by authentic standards if available. Overall, more than 80 metabolites in the liver, feces, serum, and urine were identified as either *Scenedesmus*-derived or *Scenedesmus*-responsive metabolites (Table 1). Based on their origins and biochemical properties, these metabolite markers were further categorized as the indicators of *Scenedesmus* exposure, and *Scenedesmus*-induced changes in amino acid, nucleotide, lipid, antioxidant, and microbial metabolism.

### 3.4. Scenedesmus Exposure Markers

**Figure 4:**
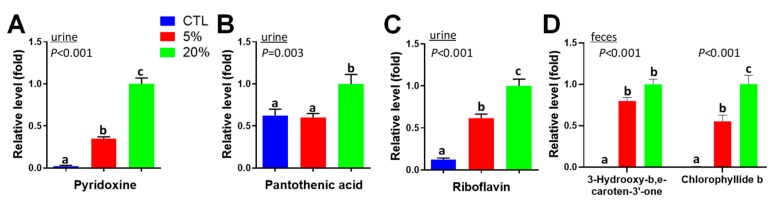
Vitamins and pigments as *Scenedesmus* exposure markers in urine and feces. (**A**) Pantothenic acid in urine. (**B**) Pyridoxine in urine. (**C**) Riboflavin in urine. (**D**) 3-Hydroxy-b,e-caroten-3′-one and chlorophyllide b in feces. *P*-values indicate overall significances across all sample groups from the one-way ANOVA test. Means with different letter labels (a,b,c) indicate significant differences (*P* < 0.05) between two dietary treatments by the Tukey post hoc test.

Water-soluble B vitamins, including pyridoxine (B6), riboflavin (B2), and pantothenic acid (B5), as prominent urinary metabolites increased by *Scenedesmus* (Figure 3F). Among them, pyridoxine and riboflavin were increased by *Scenedesmus* in a dose-dependent manner, while pantothenic acid (B5) was only increased by feeding the 20% *Scenedesmus* diet (Figure 4A–C). To determine whether the elevated urinary excretion of B vitamins was correlated with the concentrations inside the body, the distribution of riboflavin and its metabolites flavin mononucleotide (FMN) and flavin adenine dinucleotide (FAD) in the liver were examined, which showed no significantly changes by *Scenedesmus* feeding (Supplementary Figure S1), suggesting that extra riboflavin was not effectively retained in the body. In addition, prominent changes in color were visible in fecal samples from feeding the *Scenedesmus* diets. Two pigments, 3-hydroxy-b,e-caroten-3′-one, a degradation product of carotenoids, and chlorophyllide b, a component of algal chloroplast, were largely absent in control fecal samples, but present in the fecal samples from mice fed *Scenedesmus* in a dose-dependent manner (Figure 4D). Overall, these results suggest that vitamins and phyto-pigments can function as effective exposure markers of *Scenedesmus* feeding.

### 3.5. Effects of Scenedesmus Feeding on Amino Acid Homeostasis

To determine whether increased consumption of *Scenedesmus* protein could affect the homeostasis of amino acids (AA) in mice, the concentrations of free amino acids (FAAs) in the liver and serum were quantified. The results indicated that protein enrichment in *Scenedesmus* diets was not translated into the increases of FAAs because total concentrations of FAAs in serum and the liver were comparable among the three dietary treatment groups of mice (Table 2). However, selective influences on individual AA were observed after four weeks of *Scenedesmus* feeding (Table 2). Taurine, a nonproteinogenic AA, was consistently decreased by *Scenedesmus* feeding in both serum and liver, while aspartic acid and glycine were increased by 5% and 20% diet, respectively. Amino acids involved in the urea cycle were also changed by *Scenedesmus* feeding: Arginine and citrulline concentrations were increased by feeding the 20% *Scenedesmus* diet in both serum and liver, while the concentration of ornithine in serum was reduced by feeding the 20% diet. In addition, serum concentrations of essential AA, including lysine and threonine, were decreased by *Scenedesmus* feeding, while the level of methionine was elevated by feeding the 20% diet (Table 2).

### 3.6. Effects of Scenedesmus Feeding on Purine Metabolism

**Figure 5:**
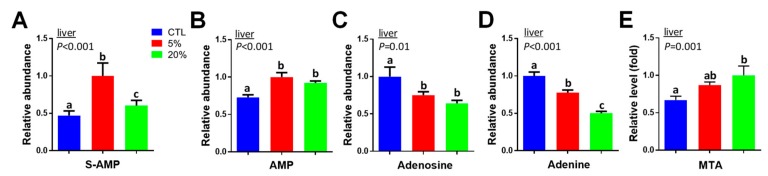
Effects of *Scenedesmus* feeding on purine metabolism. (**A**) Adenylosuccinate (S-AMP) in the liver. (**B**) Adenosine monophosphate (AMP) in the liver. (**C)** Adenosine in the liver. (**D**) Adenine in the liver. (**E**) 5’-Methylthioadenosine (MTA) in the liver. *P*-values indicate overall significances across all sample groups from the one-way ANOVA test. Means with different letter labels (a,b,c) indicate significant differences (*P* < 0.05) between two dietary treatments by the Tukey post hoc test.

In the PLS-DA model of the hepatic metabolome, multiple metabolites from purine metabolism were identified as *Scenedesmus*-responsive metabolites (Figure 3B). Among these metabolite markers, the levels of adenylosuccinate (S-AMP) and adenosine monophosphate (AMP) were increased by *Scenedesmus* feeding, especially for the 5% diet inclusion rate (Figure 5A,B). Adenosine and adenine were dose-dependently decreased by *Scenedesmus* feeding (Figure 5C,D), while 5′-methylthioadenosine (MTA) was dose-dependently increased (Figure 5E).

### 3.7. Effects of Scenedesmus Feeding on the Lipidome

**Figure 6:**
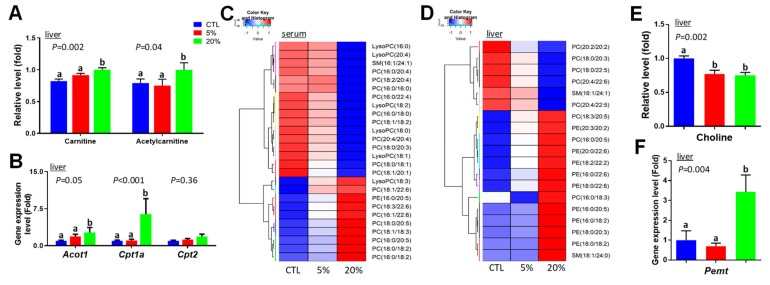
Identification of lipid metabolism-associated changes in serum and liver after *Scenedesmus* feeding. (**A**) Hepatic carnitine-related metabolites. *(***B**) Hepatic gene expression levels of PPARα-targeted genes. The control is artificially set as 1. *(***C**) Hierarchal cluster analysis (HCA)-based heat map on the clustering of *Scenedesmus*-responsive phospholipids (PL) in serum based on their relative abundances. *(***D**) HCA-based heat map on the clustering of *Scenedesmus*-responsive PL in the liver based on their relative abundances. (**E**) Choline in the liver. **F**. Expression level of *Pemt* gene in the liver. *P*-values indicate overall significances across all sample groups from the one-way ANOVA test. Means with different letter labels (a,b) indicate significant differences (*P* < 0.05) between two dietary treatments by the Tukey post hoc test.

Examining the chromatograms from the LC-MS analysis of polar and neutral lipids in the liver and serum samples, revealed that feeding the 20% *Scenedesmus* diet dramatically decreased TAG levels in the liver and also in serum (Supplementary Figure S2A,B), which is consistent with the results from blood biochemical analysis (Figure 2C). Increased PUFA-containing TAG species were also observed (Supplementary Figure S2C,D). Further analysis of aqueous liver extracts showed that feeding the 20% *Scenedesmus* diet increased the levels of free carnitine and acetylcarnitine (Figure 6A). Because fatty acid oxidation is mainly regulated by peroxisome proliferator-activated receptor alpha (PPARα), the expression of PPARα target genes in the liver was examined. The results showed that the expression levels of *Acot1* and *Cpt1*, but not *Cpt2*, were elevated by feeding the 20% *Scenedesmus* diet (Figure 6B).

In addition to the changes in TAG, *Scenedesmus*-responsive phospholipids (PL) and lyso-phospholipids (lyso-PL) species in the liver and serum were identified in the PLS-DA models of the liver and serum metabolomes (Figure 3B,D), along with further clustered analysis based on their relative abundances (Figure 6C,D). Analyzing the fatty acid composition of serum PL revealed that feeding the 20% *Scenedesmus* diet increased the abundance of phosphatidylcholine (PC) and lyso-PC species containing ω-3 PUFA, including α-linolenic acid (18:3), eicosapentaenoic acid (EPA, 20:5), and docosahexaenoic acid (DHA, 22:6), but reduced the distribution of saturated and monounsaturated fatty acids (FAs), including palmitic acid (16:0), palmitoleic acid (16:1), stearic acid (18:0), and oleic acid (18:1) (Figure 6C). Moreover, feeding *Scenedesmus* increased the relative abundance of many phosphatidylethanolamine (PE) species in the liver, while decreased that of PC species, leading to a clear shift in the profile of hepatic PL (Figure 6D). Choline, the essential nutrient used in the synthesis of PC, was decreased by *Scenedesmus* feeding (Figure 6E). Subsequent qPCR analysis showed that the gene expression level of PEMT, the enzyme responsible for converting PE to PC by sequential methylation in the liver, was increased by feeding the 20% *Scenedesmus* diet (Figure 6F).

### 3.8. Effects of Scenedesmus Feeding on Redox Balance

**Figure 7:**
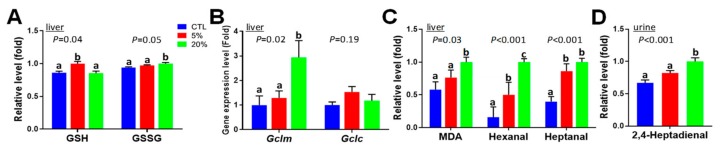
Effects of *Scenedesmus* feeding on hepatic and urinary metabolites associated with redox status. (**A**) Reduced glutathione (GSH) and oxidized glutathione (GSSG) in the liver. (**B**) Expression levels of *Gclm* and *Gclc* genes in the liver. The control is artificially set as 1. (**C**) Aldehydic lipid oxidation products (LOPs) in the liver. (**D**) 2,4-Heptadienal in urine. *P*-values indicate overall significances across all sample groups from the one-way ANOVA test. Means with different letter labels (a,b,c) indicate significant differences (*P* < 0.05) between two dietary treatments by the Tukey post hoc test.

Within the markers contributing to the separation of treatment groups in the PLS-DA model of hepatic metabolome, reduced glutathione (GSH) was positively correlated with feeding the 5% *Scenedesmus* diet, while oxidized glutathione (GSSG) was positively correlated with feeding the 20% *Scenedesmus* diet (Figure 3B). Subsequent quantitative analysis confirmed that 5% *Scenedesmus* diet increased hepatic GSH concentration, while feeding the 20% increased hepatic GSSG concentration (Figure 7A and Supplementary Figure S3). Examining the gene expression levels of the modifier and catalytic units of glutamate cysteine ligase (*Gclm* and *Gclc*), the rate limiting enzyme in glutathione biosynthesis showed that feeding the 20% *Scenedesmus* diet induced *Gclm* gene expression, while *Gclc* expression was not affected (Figure 7B). Moreover, aldehydic lipid oxidation products (LOPs), including malondialdehyde (MDA), hexanal and heptanal in the liver, and 2,4-heptadienal in urine, were increased by feeding *Scenedesmus*, especially from 20% diet inclusion rate (Figure 7C,D). All of these observations related to thiol antioxidants and LOPs suggest that redox balance was affected by feeding *Scenedesmus* in a dose-dependent manner.

### 3.9. Effects of Scenedesmus Feeding on Microbial Metabolism

**Figure 8:**
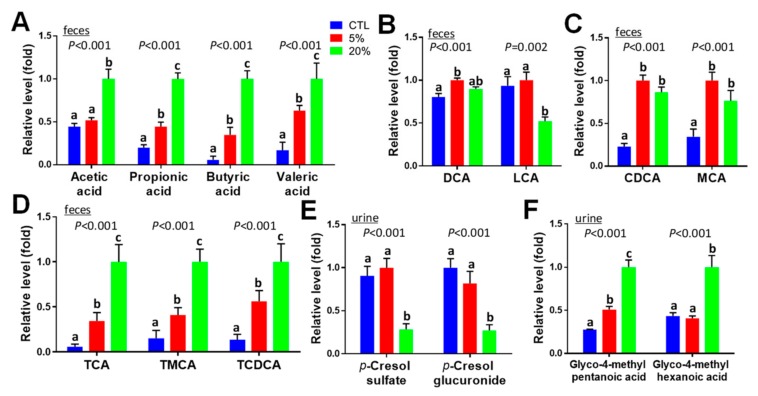
Effects of *Scenedesmus* feeding on microbial metabolites. (**A**) Short chain fatty acids (SCFAs) in feces. (**B**) Secondary bile acids in feces. (**C**) Primary bile acids in feces. (**D**) Taurine-conjugated bile acids in feces. (**E**) *p*-cresol metabolites in urine. (**F**) Glycine conjugates of branched fatty acids in urine. *P*-values indicate overall significances across all sample groups from the one-way ANOVA test. Means with different letter labels (a,b,c) indicate significant differences (*P* < 0.05) between two dietary treatments by the Tukey post hoc test.

Influences of *Scenedesmus* feeding on microbial metabolism were reflected by the metabolite markers contributing to the separation of three dietary treatments in the PLS-DA models of fecal and urine metabolomes (Figure 3H,F). Short-chain fatty acids (SCFAs) and bile acids are two types of metabolites in feces that were dramatically affected by feeding *Scenedesmus*. Among SCFAs, propionic acid, butyric acid and valeric acid were dose-dependently increased by feeding *Scenedesmus* (Figure 8A and Supplementary Figure S4). Among secondary bile acids that are formed by the microbial metabolism of unabsorbed primary bile acids, deoxycholic acid (DCA) was increased by feeding the 5% *Scenedesmus* diet, while lithocholic acid (LCA) was decreased by the 20% (Figure 8B). Levels of chenodeoxycholic acid (CDCA) and muricholic acid (MCA), two major primary bile acids in mouse, were increased in both *Scenedesmus* treatments compared with feeding the control diet (Figure 8C). Moreover, taurine-conjugated bile salts, including taurocholic acid (TCA), tauromuricholic acid (TMCA), and taurochenodeoxycholic acid (TCDCA), were dramatically and dose-dependently increased by *Scenedesmus* feeding (Figure 8D). In urine samples, *p*-cresol sulfate and *p*-cresol glucuronide derived from the microbial degradation of tyrosine were decreased by feeding the 20% *Scenedesmus* diet (Figure 8E), while glyco-4-methyl-pentanoic acid and glyco-4-methyl-hexanoic acid, two metabolites that might be derived from bacterial branched fatty acids were increased (Figure 8F).

## 4. Discussion

**Figure 9:**
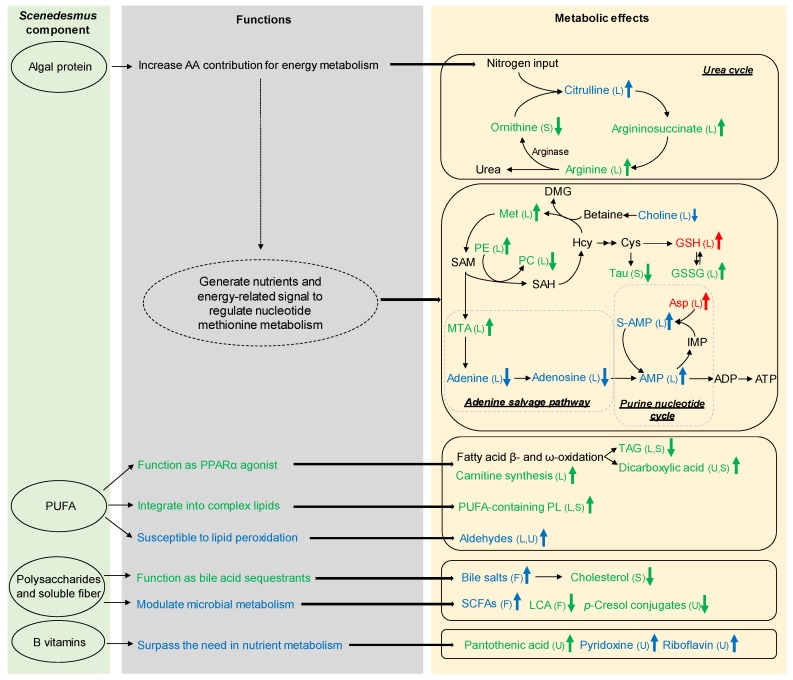
Summary on *Scenedesmus*-induced metabolic effects and its potential role in modulating metabolic system. Variety of components in *Scenedesmus* exert different functions in the body, which contribute to the metabolic changes after *Scenedesmus* feeding. Overall, *Scenedesmus* feeding targets multiple metabolic system, including urea cycle, purine metabolism, methionine metabolism, nuclear receptor PPARα metabolic pathway, redox balance, and microbial metabolism. Metabolic effects only induced by 5% are presented in red color, whereas 20% are in green color. Blue color indicate the metabolic effects induced by both 5% and 20% feeding. (AA, amino acid; Met, methionine; Hcy, homocysteine; Cys, cysteine; Tau, taurine; SAM, s-adenosylmethionine; SAH, s-adenosylhomocysteine; DMG, dimethylglycine; Asp, aspartic acid; ADP, adenosine diphosphate; ATP, adenosine triphosphate).

Macronutrient, micronutrient, and secondary metabolite content of microalgae, including fatty acids, metals, and allelopathic compounds, are greatly different from that of common plant- and animal-derived feed ingredients [16]. These compositional differences are expected to cause differences in the nutritional, physiological, and metabolic responses of humans and animals when a significant amount of microalgae is consumed. In these feeding-induced changes, algae-derived and algae-responsive metabolites can function as initiators, executors, or end-products in various metabolic processes. In the current study, the metabolites affected by feeding *Scenedesmus* were identified and characterized through biochemical and metabolomic analysis. The discussion of these metabolites is primarily based on their biochemical, physiological, and metabolic functions, as well as their associations with the chemical and nutritional properties of *Scenedesmus* components and the dose-dependent effects of *Scenedesmus* feeding on growth (Figure 9).

### 4.1. Causes and Significance of Scenedesmus-Induced Changes in Vitamins

Microalgae contain diverse cofactors needed for autotrophic metabolic activities [17]. Some of these cofactors are essential vitamins required by humans and animals. Except cobalamin (B12), thiamine (B1), and biotin (B7), microalgae are capable of synthesizing the majority of B vitamins, including riboflavin (B2), pyridoxine (B6), and pantothenic acid (B5), as observed in this study [18,19]. Because these water-soluble vitamins are mainly eliminated through urinary excretion, the elevation of their levels in urine of *Scenedesmus*-fed mice was not surprising. However, extra riboflavin intake from consuming *Scenedesmus* diets did not lead to the increases of riboflavin, FMN, and FAD in the mouse liver, suggesting that *Scenedesmus*-derived riboflavin likely exceeded the daily requirement; and therefore, was not retained by the body, partially due to its water solubility [20]. Interestingly, dephospho-CoA, a pantothenic acid derivative and also an immediate precursor of coenzyme A, was identified as a metabolite in the hepatic metabolome that was positively correlated with the 5% feeding (Table 1). Therefore, it is plausible that the utilization of *Scenedesmus*-derived pantothenic acid for coenzyme A biosynthesis was increased by the 5% feeding. Overall, the supplementation of *Scenedesmus* to the vitamin-sufficient AIN93G diet in this study may have limited impacts on the status of B vitamins inside the body.

### 4.2. Causes and Significance of Scenedesmus-Induced Changes in Nitrogen Metabolism

Despite the increase of protein content from 197 g/kg in the control diet to 263 g/kg in the 20% *Scenedesmus* diet (Table S2), *Scenedesmus* supplementation did not increases the size of the total FAAs pool in the liver and serum (Table 2). Because microalgae, in general, have a lower level of carbohydrate content than common plant-derived ingredients (such as 10% to 17% in *Scenedesmus obliquus* vs. 30% in soybean) [21], the additional amino acids derived from the proteins in *Scenedesmus* were likely utilized to compensate for the decrease of carbohydrates in the diet for use in energy metabolism. This shift in macronutrient metabolism requires the deamination of amino acids and may increases the volume of nitrogen metabolism (Figure 9). The changes of urea cycle-related metabolites including FAAs, dimethylarginine (liver), and argininosuccinate (liver) (Supplementary Figure S5) indicate that feeding the 20% *Scenedesmus* diet can significantly disrupt urea cycle after four weeks of consumption, potentially through the negative regulation on arginase (Figure 9). Interestingly, dimethylarginine is also known as an inhibitor of arginase [22] and nitric oxide synthase (NOS) [23]. Whether this event contributes to the inhibitory effect of consuming the 20% *Scenedesmus* diet on urea cycle requires further studies.

### 4.3. Causes and Significance of Scenedesmus-Induced Changes in Purine Nucleotide Metabolism

In addition to serving as the building blocks of the genome, purine nucleotides are important components in energy metabolism by functioning as energy carriers and regulatory cofactors. The alteration of purine nucleotide metabolism is implicated by the changes of multiple metabolites in purine nucleotide cycle (PNC) (Figure 9). Because PNC plays an important role in sustaining ATP production and aerobic energy metabolism [24], the increases of aspartic acid, S-AMP, and AMP, especially when feeding the 5% *Scenedesmus* diet, suggests an association of enhanced PNC activity and elevated energy production with the improved growth performance. Interestingly, hepatic MTA, a sulfur-containing nucleoside that is produced during the biosynthesis of polyamines, was increased by feeding the 20% *Scenedesmus* diet (Figure 5E). The conversion of MTA to adenine plays a critical role in salvaging adenine for AMP synthesis when de novo purine synthesis is not active [25]. Therefore, the observation of opposite changes in MTA and adenine when mice were fed the 20% *Scenedesmus* diet suggests a suppression of adenine salvage, which may negatively affect energy metabolism. Because nucleotide metabolism is extensively regulated by nutrients [26], the macronutrient composition of 5% and 20% *Scenedesmus* diets likely contributed to these dose-dependent changes in purine metabolites. Further studies are required to reveal the underlying mechanisms of these responses.

### 4.4. Causes and Significance of Scenedesmus-Induced Changes in the Lipidome and Lipid Metabolism

Microalgae feeding is expected to affect the lipidome because the unique composition of algal lipids, especially the enrichment of PUFAs, is a major cause behind their usage as dietary supplements [17]. *Scenedesmus* sp. in this study contains elevated levels of PUFAs, especially EPA [11]. PUFAs and their metabolites are the known ligands of peroxisome proliferator-activated receptors (PPARs), including PPARα and PPARγ [27], and are also the active components in fish oil contributing to their lipid modulation effects [28]. The upregulation of PPARα targeted genes in this study is consistent with the known bioactivities of PUFAs. As the direct targets of PPARα activation, fatty acid β-oxidation and ω-oxidation appear to be induced by feeding the 20% *Scenedesmus* diet, resulting in the decreases of serum and hepatic TAG and the increases of dicarboxylic acids in urine and serum (Table 1), respectively. Additionally, the increases of carnitine and short-chain acylcarnitine could also be the consequence of PPARα activation, because both carnitine biosynthesis and transport are positively regulated by PPARα [29], and short-chain acylcarnitines were decreased in PPARα-null mice [30]. Furthermore, because lysine is the precursor metabolite of carnitine [31], it is also possible that increased carnitine biosynthesis could contribute to the decrease of lysine in serum. In addition to the activation of PPARα, PUFAs can suppress the expression of sterol regulatory element-binding protein-1 (SREBP-1) [32], the transcriptional regulator of cholesterol biosynthesis, which may contribute to the decreased serum cholesterol level observed when feeding the 20% *Scenedesmus* diet (Figure 2D). The dose-dependent increases of taurine bile salts in feces (Figure 8D) indicate that soluble fiber contents in *Scenedesmus* may function as bile acid sequestrants to achieve hypolipidemic effects through preventing the reabsorption of bile acids in the small intestine [33].

In addition to TAG and cholesterol, feeding the 20% *Scenedesmus* diet also affected the composition of PL in the liver and serum. The decrease of PC/PE ratio has been commonly defined as an indicator of PEMT activity based on the phenotype of *Pemt* knockout mice, in which a significant decrease in PC content occurred in the liver, with or without choline deficiency [34]. However, the observations in current study share some similarity with the previously reported effects of feeding choline-deficient diets, which decreased the PC:PE ratio, but also induced the expression of *Pemt* in rats [35]. Decreased hepatic choline level may have been caused by decreased choline intake because algae usually contain less choline than common plant and animal sources of feed ingredients [36]. Further studies are required to determine the underlying mechanism behind these observations. Given the fact that the PC:PE ratio determines the chemical and physical properties, as well as the integrity of mammalian membranes [37], the biological functions of PL membranes in the mouse liver may be negatively affected by feeding the 20% *Scenedesmus* diet in this study. Interestingly, choline supplementation to laying hens fed a microalgae (*Schizochytrium*) diet was effective in improving the incorporation of PUFAs into PC and Lyso-PC content of egg yolk [38].

### 4.5. Causes and Significance of Scenedesmus-Induced Changes in Redox Balance

GSH is the critical thiol antioxidant and reliable indicator of redox status [39]. In this study, the redox homeostasis was clearly affected by *Scenedesmus* feeding in a dose-dependent pattern. The disruption of the thiol antioxidant system in the 20% *Scenedesmus* dietary treatment was also supported by other two lines of evidence: (1) The imbalance between methionine and taurine suggests a disruption of the metabolic pathways between them because methionine is the precursor of thiol antioxidants, while taurine is the end product of transsulfuration and cysteine oxidation. (2) The induction of *Gclm* expression by the 20% *Scenedesmus* diet, which is a rate-limiting enzyme in GSH biosynthesis [40], is a characteristic event indicating the upregulation of the transcriptional activity of nuclear factor erythroid 2-related factor 2 (Nrf2) in response to oxidative stress signals [41]. Based on the observation that multiple LOPs were increased by feeding the 20% *Scenedesmus* diet (Figure 7C,D), as well as the fact that ω-3 PUFA are the major precursors of these aldehydes through peroxidation [42], the PUFAs content in *Scenedesmus* may be a contributing source of oxidative stress when added to mouse diets at a 20% inclusion rate (Figure 9).

### 4.6. Causes and Significance of Scenedesmus-Induced Changes in Microbial Metabolism

Microalgae are a rich source of prebiotic carbohydrates, mainly due to their polysaccharide and soluble fiber content [17,43]. *Scenedesmus*, together with other microalgae, such as *Chlorella* species, are rich in polysaccharides [21]. The prebiotic functions of *Scenedesmus* ingredients in this study were reflected by the changes in fecal microbial metabolites, including SCFAs, bile acids, and *p*-cresol (Figure 9). The observation of dramatic dose-dependent increases of fecal SCFAs in this study clearly suggests that the polysaccharides in *Scenedesmus* are highly fermentable for producing SCFAs, which can provide the fuel for colonocyte proliferation and energy metabolism as well as regulate metabolism and immune responses through their signaling functions [44]. In addition to polysaccharides and soluble fiber, *Scenedesmus*-derived PUFAs may also play a role in promoting SCFAs production, since it has been shown that high content of PUFAs in the diet may lead to an elevated colonic fermentation [45].

Gut microbiota-mediated formation of secondary bile acids is a two-step process, in which primary bile acids (CDCA and MCA) are formed by the deconjugation of taurine or glycine moieties from primary bile salts (TCA, TCDCA, and TMCA), and are then subsequently converted to secondary bile acids (DCA and LCA) through dehydroxylation [46]. Based on the ratios of primary bile acid:primary bile salt and secondary bile acid:primary bile acid in this study (Figure 8B–D), it appears that feeding the 5% *Scenedesmus* diet mainly inhibited the primary bile acid:secondary bile acid step, while feeding the 20% *Scenedesmus* diet negatively affected both steps. Results from a previous study showed that fiber can decrease the activities of microbial enzymes responsible for converting primary bile acids to secondary bile acids [47]. This mechanism, along with the well-known modulating effects of fiber on the microbiome, may explain the observed dose-dependent effects of *Scenedesmus* on the microbial metabolism of bile acids. In addition, the inhibitory effects of feeding a diet containing 20% *Scenedesmus* on the formation of *p*-cresol, a microbial metabolite of tyrosine, is consistent with the reported inhibitory effect of fiber on *p*-cresol formation [48]. Overall, these microbial metabolite data warrant further investigation on the dose-dependent effects of *Scenedesmus* feeding on the microbiome and microbial metabolism.

## 5. Conclusions

Overall, *Scenedesmus* offers a combination of protein, lipids, vitamins, fiber, and bioactive constituents that are significantly different from common plant or animal feed ingredients. These compositional differences in nutrients and non-nutrients led to diverse and dramatic changes in the mouse metabolome observed in this study. The growth promotion effect from feeding the 5% *Scenedesmus* diet was associated with elevated antioxidants, an expanded purine nucleotide cycle, and modified microbial metabolism, while the growth suppression effect observed when feeding the 20% *Scenedesmus* diet was correlated to oxidative stress, disrupted urea cycle, upregulated fatty acid oxidation, and an imbalanced lipidome. These dose-dependent effects clearly suggest the existence of an inclusion threshold for the dietary addition of *Scenedesmus* as an animal feed ingredient. Further studies on the metabolic fates of individual algal constituents, as well as their influences on specific metabolic routes, such as purine, methionine, PUFA, and SCFA metabolism, will provide useful insights on the chemical signals and biological pathways contributing to dose-dependent beneficial and adverse effects, and guide the uses of *Scenedesmus* and also other microalgae as animal feed ingredients.

## Figures and Tables

**Table 1:** Effects of *Scenedesmus* feeding on hepatic, serum, urinary, and fecal metabolomes *.

	Markers of CTL Diet	Markers of 5% Diet	Markers of 20% Diet
**Liver**	Adenione ^1^, adenine ^1^, choline ^1^, nicotinamide ^1^	Glutathione (GSH) ^1^, dephospho-CoA ^2^, adenylosuccinate (S-AMP) ^1^, AMP ^1^	Oxidized glutathione (GSSG) ^1^, dimethylarginine ^1^, carnitine ^1^, acetylcarnitine ^1^, 3-dehydroxycarnitine ^3^, glutarylcarnitine ^3^, glycerylphosphoethanolamine ^3^, 5′-methylthioadenosine (MTA) ^1^, malondialdehyde (MDA) ^3^, heptanal ^1^, hexanal ^1^
**Serum**	Palmitoleic acid ^3^	Glutamate ^1^, suberic acid ^1^	Glycine ^1^, arginine ^1^, sebacic acid ^1^, capric acid ^3^
**Urine**	*p*-Cresol sulfate ^2^, *p*-cresol glucuronide ^2^, glycine ^1^, phenylalanine ^1^, pyroglutamate ^1^,indolelactic-acid ^3^, 3-oxo-4-pentenoic acid ^3^		Riboflavin ^1^, pyridoxine ^1^, pantothenic acid ^1^, glycol-4-methyl pentanoic acid ^1^, glycol-4-methyl hexanoic acid ^1^, heptenal ^1^, sebacic acid ^1^, capric acid ^3^
**Feces**	Indole-3-carboxylic acid ^1^, coprocholic acid ^3^, hydrocinnamic acid ^3^, eicosatrienoic acid ^3^, 3-hydroxy-hexadecanoic acid ^3^, 7-ketodeoxycholic acid ^3^	Muricholic acid (MCA) ^1^, deoxycholic acid (DCA) ^1^, lithocholic acid (LCA) ^1^, *N*-acetylhistamine ^3^	α-Linolenic acid ^1^, linoleic acid ^1^, oleic acid ^1^, tauromuricholic acid (TMCA) ^1^, taurochenodeoxycholic acid (TCDCA) ^1^, taurocholic acid (TCA) ^1^, acetic acid^1^, butyric acid ^1^, propionic acid ^1^, valeric acid ^1^, 3-hydroxy-b,e-caroten-3′-one ^3^, chlorophyllide b ^3^

* The metabolite markers of each diet were identified through their positive correlations with the diet in the PLS-DA models of LC-MS data. **^1^** Metabolites were confirmed with authentic standards. ^2^ Metabolites were confirmed by MSMS fragmentation. ^3^ Metabolites were discovered based on database search.

**Table 2:** Concentrations of free amino acids (FAAs) in serum and liver.

	Serum (μM)				Liver (μg/g Tissue)			
	CTL	5%	20%	*P*-Value	CTL	5%	20%	*P*-Value
Ala	209.35 ± 33.23	236.3 ± 86.33	235.01 ± 44.69	0.599	210.22 ± 34.46	222.46 ± 35.82	186.01 ± 39.50	0.154
Arg *	189.76 ± 47.01 ^ab^	169.36 ± 66.64 ^a^	251.15 ± 43.27 ^b^	0.037	4.42 ± 0.16 ^a^	4.58 ± 0.09 ^a^	4.94 ± 0.21 ^b^	<0.001
Asn	59.06 ± 14.85	63.24 ± 37.03	66.88 ± 32.66	0.872	23.02 ± 5.69	23.60 ± 5.22	22.25 ± 4.77	0.875
Asp	16.18 ± 2.60 ^ab^	19.06 ± 4.15 ^b^	14.06 ± 1.83 ^a^	0.012	58.09 ± 9.40 ^a^	73.69 ± 19.44 ^b^	55.07 ± 16.09 ^ab^	0.051
Cit	63.85 ± 15.44 ^ab^	59.09 ± 27.04 ^a^	87.03 ± 18.62 ^b^	0.032	5.56 ± 3.09 ^a^	4.02 ± 0.96 ^b^	9.31 ± 2.13^b^	0.000
Gln	624.04 ± 87.01	612.60 ± 115.57	655.91 ± 69.84	0.632	1025.03 ± 226.17	1078.73 ± 388.07	976.34 ± 247.78	0.789
Glu	88.73 ± 12.19	101.84 ± 23.28	76.75 ± 16.16	0.065	354.49 ± 95.59	437.33 ± 121.75	310.89 ± 130.99	0.114
Gly	302.98 ± 47.69 ^a^	325.65 ± 55.44 ^ab^	383.40 ± 77.11 ^b^	0.044	276.28 ± 61.80	266.79 ± 58.33	233.65 ± 37.91	0.272
His *	97.93 ± 17.58	104.71 ± 33.91	97.46 ± 28.02	0.840	102.56 ± 15.82	101.49 ± 35.32	93.93 ± 17.81	0.322
Ile/Leu *	120.65 ± 24.04	139.01 ± 43.68	151.53 ± 46.01	0.306	45.04 ± 11.34	50.70 ± 26.84	46.06 ± 10.59	0.601
Lys *	542.26 ± 90.24 ^a^	405.56 ± 63.32 ^b^	439.35 ± 72.62 ^b^	0.006	100.18 ± 27.85	98.27 ± 38.28	94.53 ± 21.71	0.930
Met *	25.54 ± 7.03 ^a^	35.83 ± 17.73 ^ab^	41.96 ± 11.76 ^b^	0.050	14.10 ± 3.35	13.71 ± 2.74	13.99 ± 3.95	0.972
Orn	143.29 ± 31.78 ^a^	155.24 ± 46.54 ^ab^	166.18 ± 43.12 ^b^	0.545	43.31 ± 15.75	36.58 ± 11.81	38.26 ± 11.44	0.574
Phe *	89.85 ± 20.86	99.04 ± 36.21	108.95 ± 22.25	0.393	30.97 ± 6.57	37.04 ± 16.30	34.94 ± 7.93	0.857
Pro	125.51 ± 34.56	154.58 ± 82.08	164.88 ± 42.78	0.376	50.92 ± 23.48	52.92 ± 19.28	50.95 ± 16.47	0.974
Ser	198.01 ± 30.60	210.75 ± 69.02	209.04 ± 52.15	0.873	41.01 ± 7.28	41.97 ± 17.28	44.76 ± 13.58	0.845
Tau	774.51 ± 99.48 ^a^	698.40 ± 114.27 ^a^	564.45 ± 61.28 ^b^	0.001	2844.17 ± 336.96 ^a^	2682.88 ± 451.51 ^b^	2358.50 ± 274.23 ^b^	0.036
Thr *	182.54 ± 39.22 ^a^	127.68 ± 50.76 ^b^	136.21 ± 25.90 ^ab^	0.026	44.70 ± 11.74	45.95 ± 11.41	41.53 ± 11.64	0.738
Try *	136.90 ± 19.45	142.41 ± 20.01	121.20 ± 37.15	0.776	18.08 ± 2.33	18.56 ± 4.01	17.10 ± 2.45	0.626
Tyr	121.85 ± 24.15	140.49 ± 44.45	153.90 ± 46.08	0.599	59.20 ± 15.25	65.52 ± 36.55	58.84 ± 14.24	0.827
Val *	242.94 ± 46.19	276.93 ± 112.46	270.08 ± 78.47	0.695	68.63 ± 18.98	77.47 ± 31.43	60.94 ± 14.85	0.369
Total	4376.13 ± 460.59	4298.71 ± 895.25	4451.39 ± 580.96	0.902	5245.86 ± 483.405	5404.06 ± 1108.41	4610.51 ± 713.365	0.142

Values are means ± SEM. *P*-values indicate overall significances across all sample groups from the one-way ANOVA test. Means with different superscripts (^a^ or ^b^) indicate significant differences (*P* < 0.05) from the Tukey post hoc test. *: Essential amino acids.

## Supplementary Materials

The following are available online at https://www.mdpi.com/2072-6643/11/9/1971/s1. Table S1: Chemicals, Table S2: Diet composition, Table S3: The sequences of primers used in the real-time PCR analysis of gene expression, Table S4: Information on the metabolites positively correlated with individual dietary treatments, Figure S1: The distribution of riboflavin and its metabolites in the liver after *Scenedesmus* feeding, Figure S2: Identification of lipid metabolism changes after *Scenedesmus* feeding, Figure S3: *Scenedesmus*-induced changes in levels of GSH and GSSG, Figure S4: *Scenedesmus*-induced changes in the concentrations of SCFAs in feces, Figure S5: *Scenedesmus*-induced changes in levels of metabolites in urea cycle.
