# Supplementary material for: Metabolomic Evaluation of Scenedesmus sp. as a Feed Ingredient Revealed Dose-Dependent Effects on Redox Balance, Intermediary and Microbial Metabolism in a Mouse Model
Source: Nutrients. 2019 Aug 21;11(9):1971. doi: 10.3390/nu11091971 (PMC6770930; doi:10.3390/nu11091971)

Metabolomic evaluation of *Scenedesmus* sp. as a feed ingredient revealed dose-dependent effects on redox balance, intermediary and microbial metabolism in a mouse model

Yiwei Ma[1], Wenguang Zhou[2], Paul Chen[2], Pedro E. Urriola[3], Gerald C. Shurson[3], Roger Ruan[2*], Chi Chen[1*]

[1] Department of Food Science and Nutrition, University of Minnesota, St. Paul, MN 55108; maxxx792@umn.edu (Y.M.); chichen@umn.edu (C.C.)

[2] Department of Bioproducts and Biosystems Engineering, University of Minnesota, St. Paul, MN 55108; wgzhou@ncu.edu.cn (W.Z.); chenx088@umn.edu (P.C.); ruanx001@umn.edu (R.R.)

[3] Department of Animal Science, University of Minnesota, St. Paul, MN 55108; urrio001@umn.edu (P.U.); shurs001@umn.edu (G.S.)

* Correspondence: chichen@umn.edu; Tel.: +1-612-624-7704 (C.C.); ruanx001@umn.edu (R.R.)

SUPPLEMENTARY DATA

Table S1. Chemicals. All reagents and chemicals are listed alphabetically.

Reagent	Vendor	Catalog No.
Acetonitrile (ACN)	Fisher Scientific	A955-5
n-Butanol	Sigma-Aldrich	B7906
2-2′-Dipyridyl disulfide (DPDS)	MP Biomedicals, LLC	150987
2-Hydrazinoquinoline (HQ)	Alfa Aesar	H50700
Triphenylphosphine (TPP)	Alfa Aesar	14112
**Standard**	**Vendor**	**Catalog No.**
Acetic acid	Mallinckrodt Chemicals	V193-06
Acetylcarnitine, HCl	Acros Organics	342390010
Adenine, 1H-Purin-6-amine	Ark Pharm	AK-96693
Adenosine	Alfa Aesar	A10781
Adenosine-5'-monophosphoric acid (AMP)	Alfa Aesar	L14051
Adenylosuccinic acid	Sigma	A5028
Amino acid mixture (acidic)	Sigma-Aldrich	A6407
Amino acid mixture (basic)	Sigma-Aldrich	A6282
Butyric acid	Alfa Aesar	L13189 or 36544
Carnitine	Acros	108450250
Choline chloride	US Biochemical Corp	22559
Dimethylarginine	Enzo Life Science	Alx-106-005-M005
Glutathione, oxidized	Sigma-Aldrich	G4251
Glutathione, reduced	Sigma-Aldrich	G4376
Heptaldehyde, 1-heptanal	Acros organics	120320500

Hexanal (hexaldehyde)	Alfa Aesar	A16265-22
Indole-3-carboxylic acid	Acros Organics	307610010
Lithocholic acid	Sigma	L6250-10G
4-Methyl hexanoic acid	Ultra Scientific	FLBA-022
4-Methyl pentanoic acid	Bedoukian Research	476
5'-Methylthioadenosine	Sigma-Aldrich	D5011
β-Muricholic acid	Steraloids	C1895-000
Niacinamide (Nicotinamide, niacin)	Sigma	N3376
Nicotinamide adenine dinucleotide (NAD)	Sigma-Aldrich	N7004
Octanedioic acid, suberic acid	Ark Pharm	AK-73011
Pantothenic acid	Alfa Aesar	A16609-22
Propionic acid	Alfa Aesar	L04210
2-Pyrrolidone-5-carboxylic acid (pyroglutamate)	Acros Organics	232930050
Riboflavin	Alfa Aesar	A11764
Riboflavin-5'-phosphate sodium salt dihydrate	Chem-IMPEX International	Cat#00259
Sebacic acid	TCI America	S0022
Sodium Deoxycholate	Sigma-Aldrich	D6750-10G
Sodium taurochenodeoxycholate	Sigma	T6260-25mg
Sodium taurocholate hydrate	Sigma	T4009-250mg
Sodium taurodeoxycholate hydrate	Sigma	T0875-1G
Taurocholic acid	Acros Organics	230370010
Valeric acid	TCI America	V0003

Table S2. Diet composition.

	CTL	5% *Scenedesmus*	20% *Scenedesmus*
Diet composition (g/kg)			
Scenedesmus	0	50	200
AIN93G	1000	950	800
Nutrient content (g/kg)			
Crude protein	197.6	213.2	263.4
ADF	36.7	35.2	30.6
aNDF	47.3	46.3	43.2
Calcium	5.7	5.8	6
Phosphorus	3.1	3.3	4
Magnesium	0.5	0.6	0.7
Potassium	4.3	4.4	4.7

**Calculated based on the proximate analysis of AIN93G and algae powder from Dairyland Lab, Inc*

Table S3. The sequences of primers used in the real-time PCR analysis of gene expression.

Gene	Forward primer (from 5′ to 3′)	Reverse primer (from 5′ to 3′)
Acot1	TTGCACATGCGGTGCACGAG	AGGAAAGGGTCCAGGTTCTGGG
Cpt1	ACACCACTGGCCGCATGTCAAG	TGCCGTGCTCTGCAAACATCCA
Cpt2	TGCCCAGGCTGCCTATCCCTAA	GCAGCTCCTTCCCAATGCCGTT
Pemt	TGAGCCAGCCCAAGATGGAG	TCCAGCACGCTGAAGGGAAA
Gclc	TGGCCACTATCTGCCCAATT	GTCTGACACGTAGCCTCGGTAA
Gclm	TGGAGTTCCCAAATCAGCCCC	GCAACTCCAAGGACGGAGCA
Actin	TCCATCATGAAGTGTGACGTT	TGTGTTGGCATAGAGGTCTTTACG

Table S4. Information on the metabolites positively correlated with individual dietary treatments (CTL, 5%, and 20%). The metabolites are identified in the loadings plots of PLS-DA models on liver (Figure 3B), serum (Figure 3D), urine (Figure 3F), and feces (Figure 3H) after ranking all detected MS signals based on their coefficients with each dietary treatment.

RT (min)	*m/z*	Metabolite ID*	Formula of original molecule	Formula of detected ion#	Positive correlation with treatment
		Liver			
2.42	104.1076	Choline (pos)	$C_5H_{13}NO$	$C_5H_{14}NO^+$	CTL
1.81	123.0559	Nicotinamide (pos)	$C_6H_6N_2O$	$C_6H_7N_2O^+$	CTL
2.69	136.0621	Adenine (pos)	$C_5H_5N_5$	$C_5H_6N_5^+$	CTL
2.56	146.1173	3-dehydroxycarnitine (pos)	$C_7H_{15}NO2$	$C_7H_{16}NO_2^+$	20%
2.87	162.1129	Carnitine (pos)	$C_7H_{15}NO_3$	$C_7H_{16}NO_3^+$	20%
4.63	203.1505	Dimethylarginine (pos)	$C_8H_{18}N_4O_2$	$C_8H_{19}N_4O_2^+$	20%
2.16	204.1237	Acetylcarnitine (pos)	$C_9H_{17}NO_4$	$C_9H_{18}NO_4^+$	20%
5.45	214.0980	MDA (HQ)	$C_3H_4O_2$	$C_{12}H_{12}ON_3^+$	20%
4.09	216.0636	Glycerophosphoethanolamine (pos)	$C_5H_{14}NO_6P$	$C_5H_{15}NO_6P^+$	20%
5.85	242.1656	Hexanal (HQ)	$C_6H_{12}O$	$C_{15}H_{20}N_3^+$	20%
6.19	256.1814	Heptanal (HQ)	$C_7H_{14}O$	$C_{16}H_{22}N_3^+$	20%
2.66	268.1032	Adenosine (pos)	$C_{10}H_{13}N_5O_4$	$C_{10}H_{14}N_5O_4^+$	CTL
4.34	276.0847	Glutarylcarnitine (pos)	$C_{12}H_{21}NO_6$	$C_{12}H_{22}NO_6^+$	20%
2.34	298.0970	MTA (pos)	$C_{11}H_{15}N_5O_3S$	$C_{11}H_{16}N_5O_3S^+$	20%
3.61	308.0918	GSH (pos)	$C_{10}H_{17}N_3O_6S$	$C_{10}H_{18}N_3O_6S^+$	5%
4.69	348.0711	AMP (pos)	$C_{10}H_{14}N_5O_7P$	$C_{10}H_{15}N_5O_7P^+$	5%
4.66	385.1289	S-AMP (pos)	$C_{14}H_{18}N_5O_{11}P$	$C_{14}H_{19}N_5O_{11}P^+$	5%
5.62	611.1431	GSSG (pos)	$C_{20}H_{32}N_6O_{12}S_2$	$C_{20}H_{33}N_6O_{12}S_2^+$	20%
7.59	688.1462	Dephospho-CoA (pos)	$C_{21}H_{35}N_7O_{13}P_2S$	$C_{21}H_{36}N_7O_{13}P_2S^+$	5%
3.82	780.5505	PC(16:0/20:5) (pos)	$C_{44}H_{78}NO_8P$	$C_{44}H_{79}NO_8P^+$	20%
4	782.5689	PC(16:0/20:4) (pos)	$C_{44}H_{80}NO_8P$	$C_{44}H_{81}NO_8P^+$	20%
4.21	792.5513	PE(18:0/22:6) (pos)	$C_{45}H_{78}NO_8P$	$C_{45}H_{79}NO_8P^+$	20%
4.16	834.5984	PC(18:0/22:6) (pos)	$C_{48}H_{84}NO_8P$	$C_{48}H_{85}NO_8P^+$	20%
6.09	848.7693	TG(16:1/16:0/18:1) (pos)	$C_{53}H_{98}O_6$	$C_{53}H_{102}NO_6^+$	CTL
6.5	878.8137	TG(16:1/18:0/18:0) (pos)	$C_{55}H_{104}O_6$	$C_{55}H_{108}NO_6^+$	CTL
6.51	904.8297	TG(18:0/18:1/18:1) (pos)	$C_{57}H_{106}O_6$	$C_{57}H_{110}NO_6^+$	CTL
		Serum			
2.57	201.1119	Sebacic acid (neg)	$C_{10}H_{18}O_4$	$C_{10}H_{17}O_4^-$	20%
4.73	309.0909	Glycine (DC)	$C_2H_5NO_2$	$C_{14}H_{17}N_2O_4S^+$	20%
5.38	314.2231	Capric acid (HQ)	$C_{10}H_{20}O_2$	$C_{19}H_{28}ON_3^+$	20%
4.43	381.1115	Glutamate (DC)	$C_5H_9NO_4$	$C_{17}H_{21}N_2O_6S^+$	5%
4.16	408.1705	Arginine (DC)	$C_6H_{14}N_4O_2$	$C_{18}H_{26}N_5O_4S^+$	20%
4.3	496.3395	LysoPC(16:0)	$C_{24}H_{50}NO_7P$	$C_{24}H_{51}NO_7P^+$	CTL

4.14	520.3391	LysoPC(18:2)	$C_{26}H_{50}NO_7P$	$C_{26}H_{51}NO_7P^+$	CTL
4.41	522.3559	LysoPC(18:1)	$C_{26}H_{52}NO_7P$	$C_{26}H_{53}NO_7P^+$	CTL
4.66	524.3720	LysoPC(18:0)	$C_{26}H_{54}NO_7P$	$C_{26}H_{55}NO_7P^+$	CTL
5.93	671.5730	CE(20:5) (pos)	$C_{47}H_{74}O_2$	$C_{47}H_{75}O_2^+$	20%
6.31	876.8007	TG(16:0/18:1/18:1)	$C_{55}H_{102}O_6$	$C_{55}H_{106}NO_6^+$	CTL
		Urine			
1.6	170.0804	Pyridoxine (pos)	$C_8H_{11}NO_3$	$C_8H_{12}NO_3^+$	20%
3.47	172.0961	Glycol-4-methyl pentanoic acid (pos)	$C_8H_{15}NO_3$	$C_8H_{16}NO_3^+$	20%
4.3	185.1163	3-oxodecanoic acid (neg)	$C_{10}H_{18}O_3$	$C_{10}H_{17}O_3^-$	CTL
4.42	186.1120	Glycol-4-methyl hexanoic acid (pos)	$C_9H_{17}NO_3$	$C_9H_{18}NO_3^+$	20%
3.21	187.0059	*p*-Cresol sulfate (neg)	$C_7H_8O_4S$	$C_7H_7O_4S^-$	5%
2.54	201.1119	Sebacic acid (neg)	$C_{10}H_{18}O_4$	$C_{10}H_{17}O_4^-$	20%
3.58	204.0647	Indolelactic acid (neg)	$C_{11}H_{11}NO_3$	$C_{11}H_{10}NO_3^-$	CTL
1.71	220.1170	Pantothenic acid (pos)	$C_5H_7NO_3$	$C_5H_8NO_3^+$	20%
6.23	254.1660	Heptenal (HQ)	$C_7H_{12}O$	$C_{16}H_{20}N_3^+$	20%
3.28	283.0791	*p*-Cresol glucuronide (neg)	$C_{13}H_{16}O_7$	$C_{13}H_{15}O_7^-$	CTL
4.75	309.0909	Glycine (DC)	$C_2H_5NO_2$	$C_{14}H_{17}N_2O_4S^+$	CTL
5.38	314.2231	Capric acid (HQ)	$C_{10}H_{20}O_2$	$C_{19}H_{28}ON_3^+$	20%
2.99	377.1435	Riboflavin (pos)	$C_{17}H_{20}N_4O_6$	$C_{17}H_{21}N_4O_6^+$	20%
		Feces			
8.2	151.0744	Hydrocinnamic acid (pos)	$C_9H_{10}O_2$	$C_9H_{11}O_2^+$	CTL
2.61	202.0981	Acetic acid (HQ)	$C_2H_4O_2$	$C_{11}H_{12}ON_3^+$	20%
3.06	216.1137	Propionic acid (HQ)	$C_3H_6O_2$	$C_{12}H_{14}ON_3^+$	20%
3.58	230.1292	Butyric acid (HQ)	$C_4H_8O_2$	$C_{13}H_{16}ON_3^+$	20%
4.64	244.1447	Valeric acid (HQ)	$C_5H_{10}O_2$	$C_{14}H_{18}ON_3^+$	20%
7.61	271.2265	3-Hydroxy-hexadecanoic acid (neg)	$C_{16}H_{32}O_3$	$C_{16}H_{31}O_3^-$	CTL
6.92	277.2153	α-Linolenic acid (neg)	$C_{18}H_{30}O_2$	$C_{18}H_{29}O_2^-$	20%
7.86	279.2313	Linoleic acid (neg)	$C_{18}H_{32}O_2$	$C_{18}H_{31}O_2^-$	20%
8.18	281.2457	Oleic acid (neg)	$C_{18}H_{34}O_2$	$C_{18}H_{33}O_2^-$	20%
6.3	303.1205	Indole-3-carboxylic acid (HQ)	$C_9H_7NO_2$	$C_{18}H_{15}ON_4^+$	CTL
8	305.2466	Eicosatrienoic acid (neg)	$C_{20}H_{34}O_2$	$C_{20}H_{33}O_2^-$	CTL
7.01	375.2892	LCA (neg)	$C_{24}H_{40}O_3$	$C_{24}H_{39}O_3^-$	5%
4.8	391.2858	DCA (neg)	$C_{24}H_{40}O_4$	$C_{24}H_{39}O_4^-$	5%
4.63	391.2858	CDCA (neg)	$C_{24}H_{40}O_4$	$C_{24}H_{39}O_4^-$	5%
5.7	405.2630	7-Ketodeoxycholic acid (neg)	$C_{24}H_{38}O_5$	$C_{24}H_{37}O_5^-$	CTL
5.47	407.2790	MCA (neg)	$C_{24}H_{40}O_5$	$C_{24}H_{39}O_5^-$	5%
6.91	449.3250	Coprocholic acid (neg)	$C_{27}H_{46}O_5$	$C_{27}H_{45}O^-$	CTL
4.99	498.2889	TCDCA (neg)	$C_{26}H_{45}NO_6S$	$C_{26}H_{44}NO_6S^-$	20%
5.24	514.2827	TMCA (neg)	$C_{26}H_{45}NO_7S$	$C_{26}H_{44}NO_7S^-$	20%
4.91	514.2827	TCA (neg)	$C_{26}H_{45}NO_7S$	$C_{26}H_{44}NO_7S^-$	20%

8.32	567.4202	3-hydroxy-b,e-caroten-3'-one (pos)	$C_{40}H_{54}O$	$C_{40}H_{55}O^{+}$	20%
6.57	629.2222	Chlorophyllide b (pos)	$C_{35}H_{32}MgN_4O_6$	$C_{35}H_{33}MgN_4O_6^{+}$	20%

**Metabolite ID contains the compound identity and its detection mode. (pos: positive mode; neg: negative mode, DC: dansyl chloride derivatization; HQ: 2-hydrazinoquinoline derivatization.)*

[#]Formula of detected ion is the formula of charged metabolite or derivative in the MS detection.

Figure S1. The distribution of riboflavin and its metabolites in the liver after *Scenedesmus* feeding. Data are presented as mean ± SEM. *P*-values indicate overall significances across all sample groups from the one-way ANOVA test.

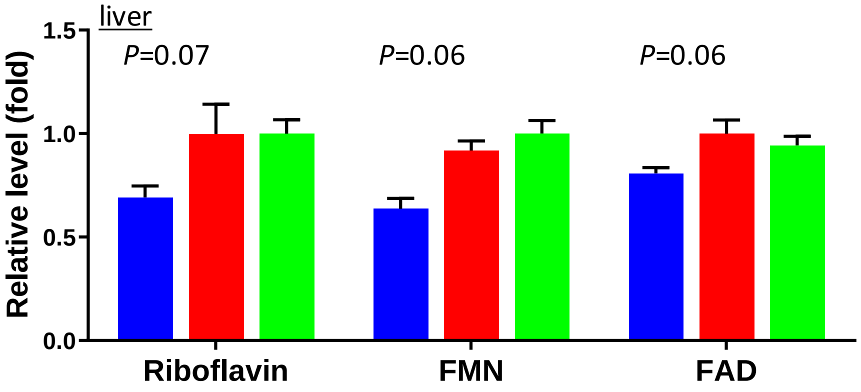

Figure S2. Identification of lipid metabolism changes after *Scenedesmus* feeding. *A.* Representative LC-MS chromatograms of extracted mouse liver lipids. ***B.*** Representative LC-MS chromatograms of extracted mouse serum lipids. ***C.*** HCA-based heat map on *Scenedesmus*–responsive TAGs in the liver. ***D.*** HCA-based heat map on *Scenedesmus*–responsive TAGs in serum.

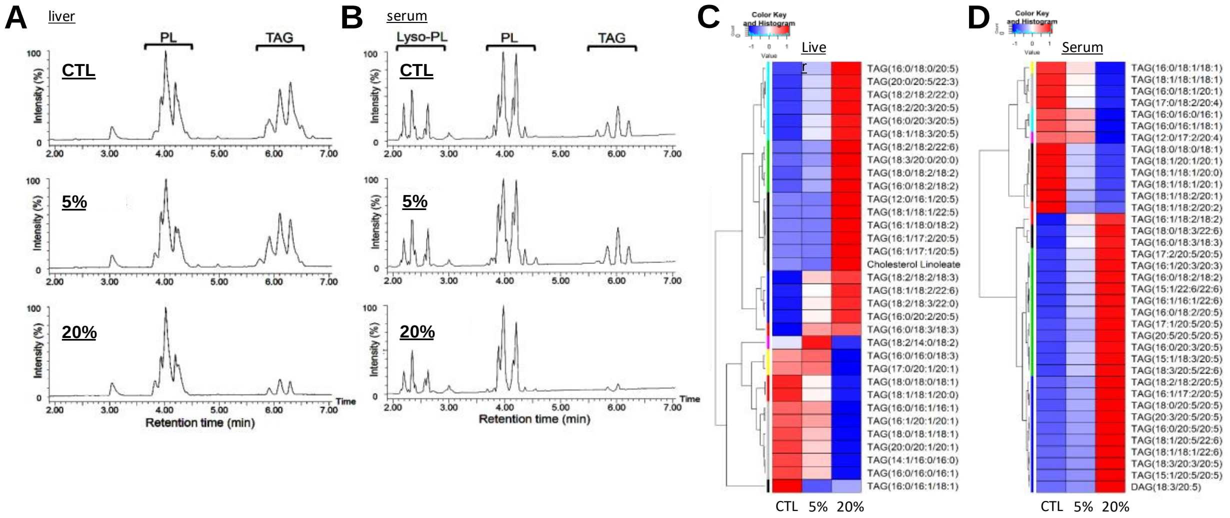

Figure S3. ***Scenedesmus*-induced changes in levels of GSH and GSSG.** A. Concentrations of GSH in the liver on day 28 of feeding. B. Concentration of GSSG in the liver. Data are presented as mean ± SEM. *P*-values indicate overall significances across all sample groups from the one-way ANOVA test. Means with different letter labels (a, b) indicate significant differences ($P < 0.05$) between two dietary treatments by the Tukey post hoc test.

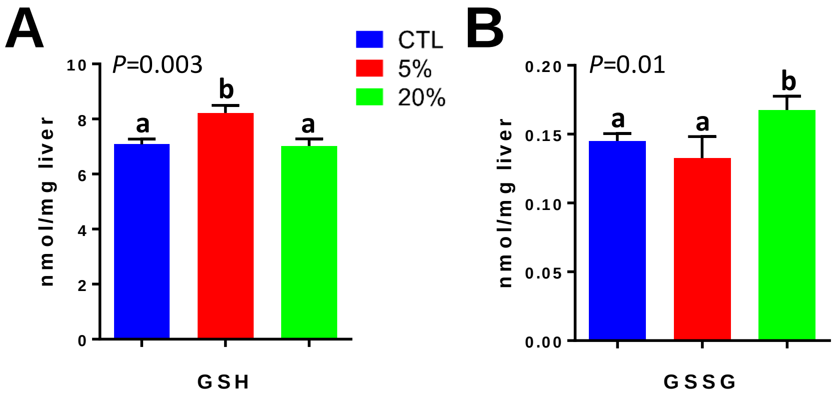

Figure S4. ***Scenedesmus*-induced changes in levels of SCFAs in feces including acetic acid, propionic acid, butyric acid and valeric acid.** Data are presented as mean ± SEM. *P*-values indicate overall significances across all sample groups from the one-way ANOVA test. Means with different letter labels (a, b, c) indicate significant differences ($P < 0.05$) between two dietary treatments by the Tukey post hoc test.

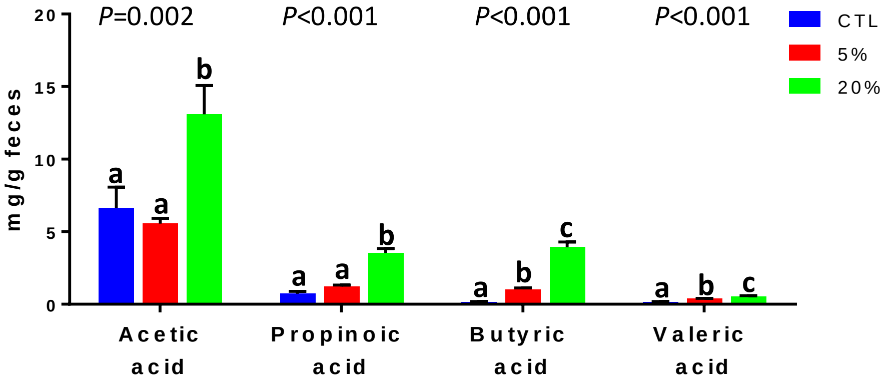

Figure S5. ***Scenedesmus*-induced changes in levels of metabolites in urea cycle.** ***A.*** Relative abundance of dimethylarginine in the liver on day 28 of feeding. ***B.*** Relative abundance of argininosuccinate in the liver. Data are presented as mean ± SEM. *P*-values indicate overall significances across all sample groups from the one-way ANOVA test. Means with different letter labels (a, b) indicate significant differences ($P < 0.05$) between two dietary treatments by the Tukey post hoc test.

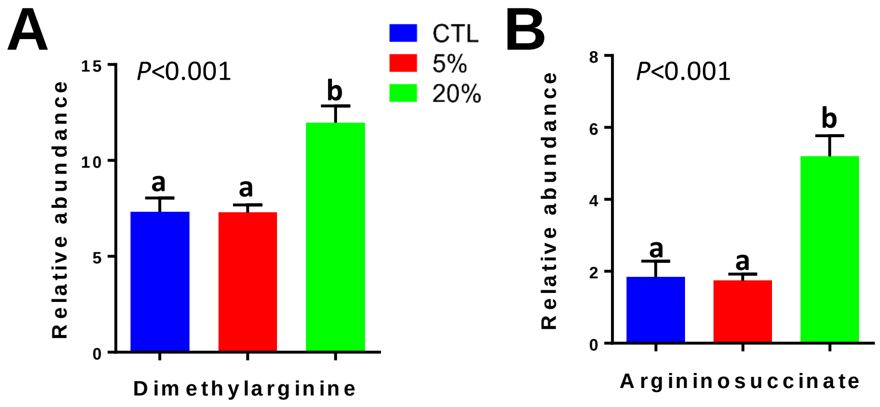

Supplement: Supplementary file 1 [file nutrients-11-01971-s001.pdf]
